# HOW TECHNOLOGY IMPACTS LONELINESS: PERSPECTIVES OF AFRICAN AMERICAN OLDER ADULTS

**DOI:** 10.1093/geroni/igad104.0823

**Published:** 2023-12-21

**Authors:** Sarah Pace

**Affiliations:** University of South Carolina, Columbia, South Carolina, United States

## Abstract

The use of information and communication technologies (ICT) has been found to decrease loneliness among older adults by enabling communication with friends and family. Over 25% of older adults experience loneliness, and having a larger social network can reduce this risk (Chawla et al., 2021; Dahlberg et al., 2022). Despite the success of using ICTs and the growing ubiquity of ICTs among people of varying social demographic backgrounds there remains a digital divide, which refers to inequality in terms of access to ICT based on race, age, economic status, or other factors. While ICT use is becoming increasingly common among older adults, few studies have included African American older adults, who are less likely to have access to high-speed internet and computers than their White counterparts. However, African American adults are more likely to use text messages, video and voice calls, email, and social media to stay connected. The proposed research aims to explore the perspectives of African American older adults on how ICT impacts loneliness. Semi-structured interviews will be used to collect data from a purposive sample of about 10 people who meet specific selection criteria. Data will be analyzed using thematic analysis. The results may be useful for gerontological practitioners interested in promoting ICT use as a way to enhance the social well-being of African American older adults. The study could lead to the development of interventions that increase technology adoption and use, ultimately reducing loneliness among African American older adults.

